# Gene-Expression-Guided Selection of Candidate Loci and Molecular Phenotype Analyses Enhance Genetic Discovery in Systemic Lupus Erythematosus

**DOI:** 10.1155/2012/682018

**Published:** 2012-07-25

**Authors:** Yelena Koldobskaya, Kichul Ko, Akaash A. Kumar, Sandra Agik, Jasmine Arrington, Silvia N. Kariuki, Beverly S. Franek, Marissa Kumabe, Tammy O. Utset, Meenakshi Jolly, Andrew D. Skol, Timothy B. Niewold

**Affiliations:** ^1^Section of Rheumatology and Gwen Knapp Center for Lupus and Immunology Research, University of Chicago, Chicago, IL 60637, USA; ^2^Section of Rheumatology and Rush Lupus Clinic, Rush University, Chicago, IL 60612, USA; ^3^Section of Genetic Medicine, University of Chicago, Chicago, IL 60637, USA

## Abstract

Systemic lupus erythematosus (SLE) is a highly heterogeneous autoimmune disorder characterized by differences in autoantibody profiles, serum cytokines, and clinical manifestations. We have previously conducted a case-case genome-wide association study (GWAS) of SLE patients to detect associations with autoantibody profile and serum interferon alpha (IFN-*α*). In this study, we used public gene expression data sets to rationally select additional single nucleotide polymorphisms (SNPs) for validation. The top 200 GWAS SNPs were searched in a database which compares genome-wide expression data to genome-wide SNP genotype data in HapMap cell lines. SNPs were chosen for validation if they were associated with differential expression of 15 or more genes at a significance of *P* < 9 × 10^−5^. This resulted in 11 SNPs which were genotyped in 453 SLE patients and 418 matched controls. Three SNPs were associated with SLE-associated autoantibodies, and one of these SNPs was also associated with serum IFN-*α* (*P* < 4.5 × 10^−3^ for all). One additional SNP was associated exclusively with serum IFN-*α*. Case-control analysis was insensitive to these molecular subphenotype associations. This study illustrates the use of gene expression data to rationally select candidate loci in autoimmune disease, and the utility of stratification by molecular phenotypes in the discovery of additional genetic associations in SLE.

## 1. Introduction

Systemic lupus erythematosus (SLE) is a severe multisystem autoimmune disease of unknown etiology. Genetic factors clearly play a role in susceptibility, and a number of genetic loci have been implicated in the disease [1]. Despite the successes of recent genetic association studies, only a fraction of the genetic liability for SLE has been explained to date. SLE is a heterogeneous disease clinically, and there is strong evidence that the molecular pathogenesis of the condition varies considerably between individuals as well. For example, specific autoantibodies are formed in some patients and not others, and these autoantibody specificities have been associated with clinical features of the disease [2, 3]. In addition, approximately half of adult patients with SLE demonstrate overactivity of the interferon alpha (IFN-*α*) pathway in their peripheral blood [2, 4]. Interestingly, high IFN-*α* and SLE-associated autoantibodies are heritable as traits in SLE families and can be found in family members who are not affected by SLE [5, 6]. Autoantibodies can be found in sera for many years prior to the clinical diagnosis of SLE [7], and it is thought that some of the autoantibodies may be themselves directly pathogenic. IFN-*α* is a cytokine involved in viral defense, capable of bridging the innate and adaptive immune systems [8]. Interestingly, when recombinant human IFN-*α* has been given as a treatment for chronic viral hepatitis, some treated individuals have developed de novo SLE, which frequently resolves upon discontinuation of the IFN-*α* [9, 10]. These data support the concept that both IFN-*α* and SLE-associated autoantibodies represent causal factors in human SLE. Additionally, both IFN-*α* and SLE-associated autoantibodies are heritable within SLE families supporting a genetic contribution, and thus the idea that these molecular measurements could be used as a phenotype in genetic studies.

In previous work, we have begun to map genetic variants which are associated with high IFN-*α* and with the presence of particular autoantibodies in SLE patients [11–13]. Some well-established genetic risk factors for SLE have been associated with one or both of these molecular phenotypes [14–18]. In addition, we have performed a genome-wide association study (GWAS) using these two molecular traits as phenotypes to enable discovery of novel genetic variants associated with IFN-*α* and SLE-associated autoantibodies [19]. A number of novel genes have been validated from this screen to date [19, 20], although much of the variance in both serum IFN-*α* and the presence or absence of particular autoantibodies remains to be explained. 

In prioritizing genetic variants to be followed up in our GWAS scan, we used gene ontogeny and expert literature search to prioritize variants which were in or near genes related to immune responses. This was based upon the supposition that SLE is an autoimmune disease, and many of the well-validated loci which have emerged from unbiased studies to date encode genes with immune function. This approach has some limitations, as genetic variations which were not near known genes were not prioritized, nor were those which did not have known function within the immune system. It is clear that genetic variants can sometimes impact the expression of a gene which is not nearby, and these genetic variants may be assigned to irrelevant nearby genes in gene ontogeny analysis. Additionally, many genes which could be critical to human disease pathogenesis may still be unstudied and unknown, and thus unlikely to be prioritized in follow up candidate studies. 

To address these possibilities in our GWAS validation, we searched our top 200 SNPs in a public database which links genome-wide SNP data from the HapMap project to genome-wide gene expression data from the HapMap lymphoblastoid B-cell lines (SCAN) database, [21]. Genes which are disease associated are more commonly associated with alternate gene expression than genes which are not disease associated [22], and thus genes from our top 200 which were strongly associated with differences in gene expression should be more likely to be true associations. In this study, we leverage gene expression data sets to prioritize additional candidates from our trait-stratified GWAS for validation in an independent cohort. We found eleven SNPs which were significantly associated with alternate gene expression of multiple transcripts in public databases, and had not been prioritized for followup in our initial GWAS screen. Four of these eleven SNPs were significantly associated with the important molecular subphenotypes IFN-*α* and SLE-associated autoantibodies in our independent validation cohort, validating this method of genetic discovery.

## 2. Methods

### 2.1. Initial GWAS Study Description

The initial cohort of SLE patients studied in the GWAS scan was obtained from the Hospital for Special Surgery Lupus Registries, and consisted of 104 SLE patients [19]. This study was designed as a case-case analysis to compare SNP frequencies in SLE patients with high versus low IFN-*α* and those with and without SLE-associated autoantibodies. Patients were selected in an extremes-of-phenotype design from the top 33% and bottom 33% of serum IFN-*α* activity and were additionally stratified for the GWAS study by ancestry and the presence or absence of anti-RBP or anti-dsDNA antibodies. A study design incorporating multiple ancestral backgrounds was chosen as both autoantibodies and serum IFN-*α* levels are heritable pathogenic factors which are shared between all ancestral backgrounds. The top 200 SNPs were examined in detail using expert review of public databases, and seven top SNPs chosen for replication using a gene-centric algorithm demonstrated strong associations with either serology or serum IFN-*α* in an independent cohort, as would have been expected based upon the initial GWAS study design [19]. 

### 2.2. Validation Cohort

The independent validation cohort of 453 SLE patients was obtained from the University of Chicago Translational Research in the Department of Medicine (TRIDOM) registry and Rush University Medical Center and consisted of 282 African-American and 171 European-American SLE patients. All patients met the revised 1982 ACR criteria for the diagnosis of SLE [23]. Samples from 418 controls were obtained from the TRIDOM registry, including 300 African-American and 118 European-American subjects who were individually screened for the absence of autoimmune disease by medical record review. The subjects in this study were not related to each other. Informed consent was obtained from all subjects at each site, and the study was approved by the IRB at each institution.

### 2.3. SCAN Database Query

We searched the top 200 SNPs from the GWAS described above as query terms in the SNP and CNV Annotation (SCAN) database (http://www.scandb.org/) [21]. This database is a searchable index of genome-wide gene expression data linked to genome-wide SNP genotype data from the HapMap project. Gene expression data is derived from studies in which gene expression arrays were run on Epstein-Barr virus-transformed lymphoblastoid cell lines from individuals genotyped in the HapMap project. The SCAN database contains expression data from both European (Centre d'Etude du Polymorphisme Humain or CEPH) and West African (Yoruba or YRI) HapMap reference populations. We used a threshold *P* value of *P* < 9 × 10^−5^ and searched both CEPH and YRI population datasets for each SNP. Because SNPs associated with alternate gene expression are more likely to be disease or trait associated [22], we selected SNPs which were associated with alternate expression of 15 or more transcripts in the SCAN database. This resulted in 11 SNPs, and for each SNP at least one of the 15 or more associated transcripts was involved in immune function. 

### 2.4. SNP Genotyping in the Validation Cohort

Individuals in the validation cohort were genotyped at the rs9521996, rs11199974, rs7785392, rs9568401, rs4892122, rs4778708, rs1340981, rs1408806, rs4894215, rs1569428, and rs1159916 SNPs. Genotyping was performed using ABI TaqMan Assays-by-Design primers and probes on an ABI 7900HT PCR machine with >98% genotyping success. Scatter plots were all reviewed individually for quality, and genotype frequencies did not deviate significantly from the expected Hardy-Weinberg proportions (*P* > 0.01 in controls across all ancestral backgrounds).

### 2.5. Reporter Cell Assay for IFN-*α*

The reporter cell assay for IFN-*α* has been described in detail elsewhere [5, 24]. Reporter cells were used to measure the ability of patient sera to cause IFN-induced gene expression. The reporter cells (WISH cells, ATCC #CCL-25) were cultured with 50% patient sera for 6 hours and then lysed. mRNA was purified from cell lysates, and cDNA was made from total cellular mRNA. cDNA was then quantified using real-time PCR using an Applied Biosystems 7900HT PCR machine with the SYBR Green fluorophore system. Forward and reverse primers for the genes *MX1*, *PKR*, and *IFIT1*, which are known to be highly and specifically induced by IFN-*α*, were used in the reaction [5]. *GAPDH* was amplified in the same samples to control for background gene expression. The amount of PCR product of the IFN-*α*-induced gene was normalized to the amount of product for the housekeeping gene *GAPDH* in the same sample. The relative expression of each of the three tested IFN-induced genes was calculated as a fold increase compared to its expression in WISH cells cultured with media alone. Results from the IFN-*α* assay were standardized to a healthy multiancestral reference population as previously described, and a serum IFN-*α* activity score was calculated based upon the mean and SD of the reference population [5]. This assay has been highly informative when applied to SLE as well as other autoimmune disease populations [5, 25–27].

### 2.6. Measurement of Autoantibodies

Antibodies to anti-Ro, anti-La, anti-Sm, and anti-RNP were measured in all samples by ELISA methods using kits from INOVA Diagnostics (San Diego, CA), and anti-dsDNA antibodies were measured using *Crithidia luciliae* immunofluorescence, with detectable fluorescence considered positive. All samples were assayed in University of Chicago clinical laboratory by the same personnel that test clinical samples. For the ELISA assays, the standard cutoff points for a positive test designated by the manufacturer were used to categorize samples as positive or negative. 

### 2.7. Statistical Analysis

**Figure 1:**
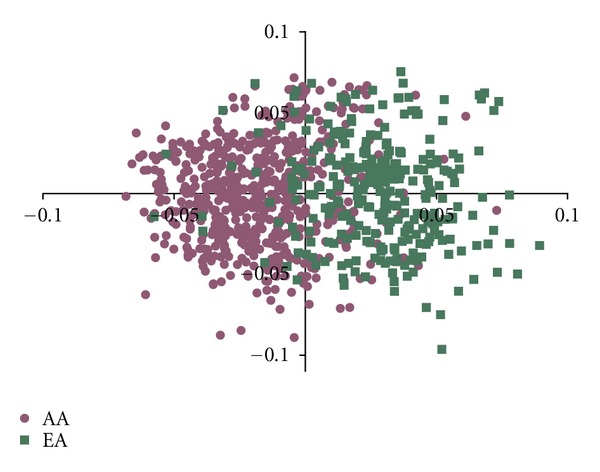
Principal component analysis of SNPs genotyped in all cases and controls. Component 1 is shown on the *x*-axis, and component 2 is shown on the *y*-axis. Each dot represents one subject, and the dots are color-coded by the self-reported ancestry of that subject.

To control for population structure and effects related to admixture, we used a principal component analysis of SNPs which varied in frequency by ancestral background. All subjects in the study had genotype data available for 30 such SNPs, and principal component analysis was performed using the PCA option in the Cluster program by Eisen et al. [28]. The first two principal components are shown plotted on the *x* and *y* axes, respectively, in Figure 1, and the first component provides a strong separation of those subjects of self-reported African-American ancestry from those of self-reported European-American ancestry. We included the first and second principal components as covariates in all subsequent association analyses to provide control for differences in proportional ancestry in both cases and controls. 

Logistic regression models were used to detect associations with SLE in case-control analysis or in case-case analyses examining the SLE-associated autoantibody traits and serum IFN-*α* activity. The SLE-associated autoantibodies anti-Ro, anti-La, anti-Sm, anti-RNP, and anti-dsDNA were all tested for association with each SNP in logistic regression models. Serum IFN-*α* was binned as high or low, using 2SD above the mean of healthy donor sera as the cutoff point, and then used as the outcome variable in logistic regression. Significant relationships observed in this regression were then explored by comparing quantitative IFN-*α* data between genotype categories. The IFN-*α* data was nonnormally distributed, and nonparametric Mann-Whitney *U* was used to compare quantitative IFN-*α* data between genotype subgroups. *P* values shown in the paper are uncorrected for multiple comparisons. To establish significance and account for multiple comparisons, we used a threshold *P* value of *P* < 4.5 × 10^−3^ to allow for a type I error rate of 0.05 following a Bonferroni correction for the number of SNPs tested in this study. 

## 3. Results

### 3.1. Three of Eleven SNPs Demonstrate Association with Autoantibody Traits in SLE Patients

**Figure 2:**
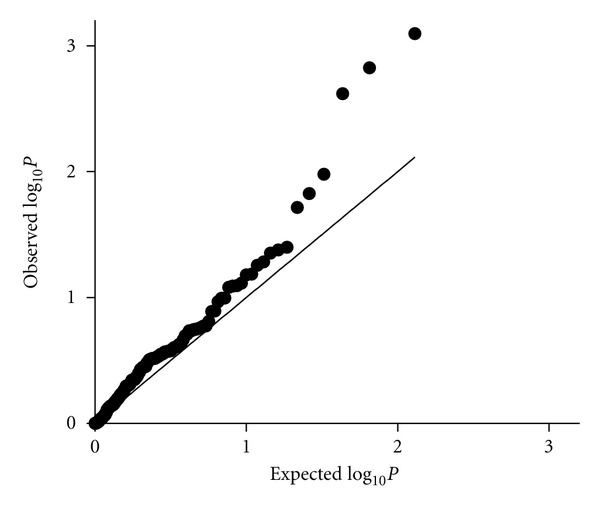
Q-Q plot showing the observed versus expected *P* values in the autoantibody analysis. *P* values that would be expected under the null hypothesis (no association between SNPs and autoantibody traits) are represented by the line, and the observed *P* values are represented by dots, one for each tested SNP-autoantibody association.

We used logistic regression to detect associations between autoantibody traits and genotype at each of the 11 SNPs in each ancestral background separately. Three SNPs demonstrated associations which would withstand a Bonferroni correction for multiple comparisons correcting for the number of SNPs tested (*P* < 4.5 × 10^−3^, Table 1).  Figure 2 shows a Q-Q plot of the distribution of probabilities observed in the antibody analyses versus the null distribution. In Figure 2, the top three SNP-antibody associations highlighted in Table 1 are represented by the three dots with the highest values on the *y*-axis which clearly deviate from the null distribution.

### 3.2. Two SNPs Are Associated with Serum IFN-*α* in SLE Patients

**Figure 3:**
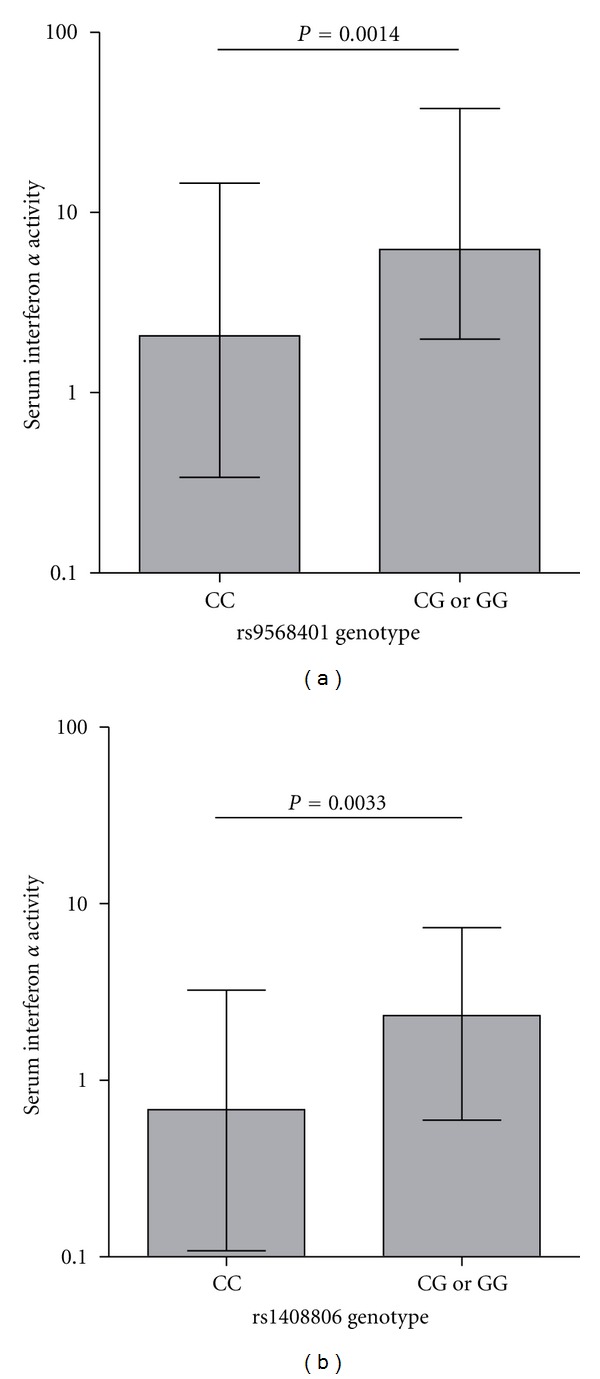
Serum IFN-*α* activity in SLE patients stratified by SNP genotype at rs9568401 (a) and rs1408806 (b). Minor allele homozygotes are combined with heterozygotes on the graph. Bars show the median error bars show the interquartile range. *P* value by Mann-Whitney *U* test.

Regression models were also used to assess the association of each of the 11 SNPs with serum IFN-*α* activity in SLE patients. An association was observed between rs9568401 G and high serum IFN-*α* in both African and European Americans. In European-Americans, the rs1408806 G allele which was associated with anti-Sm antibodies was also associated with increased serum IFN-*α*. These associations are illustrated in Figure 3, which shows quantitative IFN-*α* by genotype category. The minor allele frequency of each SNP was relatively low, and thus minor allele homozygotes were rare and are combined with heterozygotes in this graph. Dominant or recessive models could not be assessed due to the rarity of homozygous minor allele subjects, and the graph is not meant to represent a dominant relationship. While the rs1048806 SNP is also associated with an autoantibody trait, the rs9568401 SNP was not associated with any autoantibodies and was exclusively associated with serum IFN-*α* activity. 

### 3.3. Multiple Subphenotype Modeling Supports Complex Association Patterns between Genetic Variants, Autoantibodies, and Serum IFN-*α* Activity

**Figure 4:**
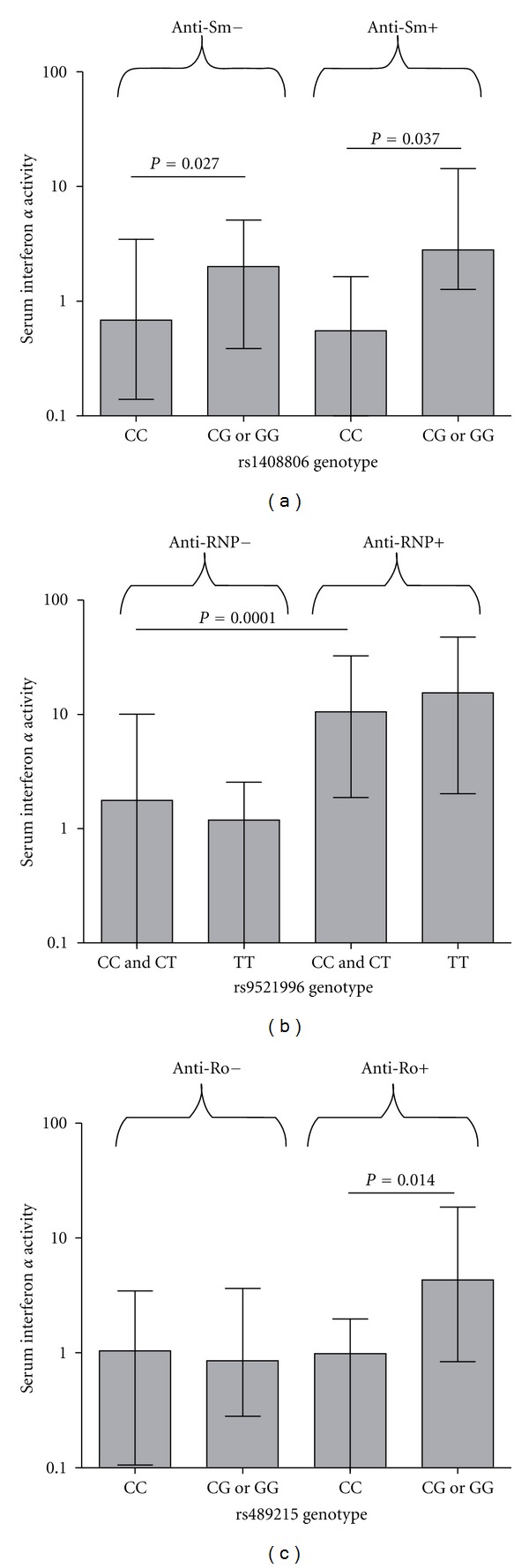
Serum IFN-*α* activity in SLE patients stratified by SNP genotype and the autoantibody associated with that particular SNP. Minor allele homozygotes are combined with heterozygotes on the graph. Bars show the median error bars show the interquartile range. *P* value by Mann-Whitney *U* test.

**Figure 5:**
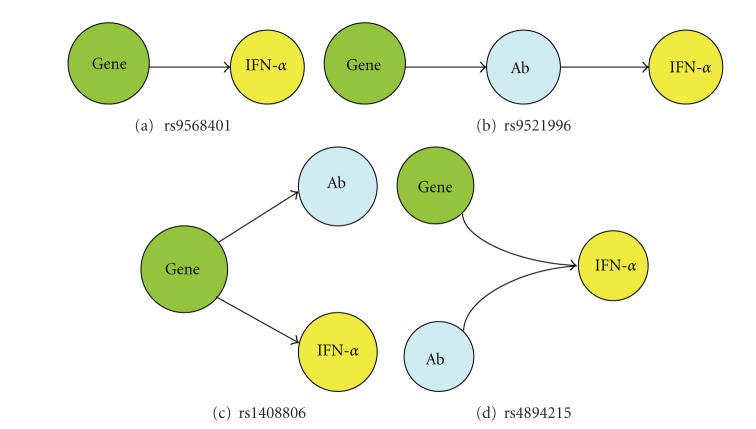
Diagrams depicting patterns of association observed between SNP genotype, autoantibodies, and serum IFN-*α*. Gene = SNP genotype at the indicated SNP, Ab = the particular autoantibody associated with that SNP, and arrows indicate the associations observed in the study.

With regard to the rs1408806 G allele which was associated with both serum IFN-*α* and anti-Sm in European ancestry, the association between these two phenotypes appeared to be independent (Figure 4(a)). Given the strong relationship between serum IFN-*α* and autoantibodies in SLE [4], we also examined serum IFN-*α* in the context of the other SNP-autoantibody relationships we had identified rs9521996/anti-RNP in African Americans and rs4894215/anti-Ro in European-Americans, (Figures 4(b) and 4(c) resp.). Both of these SNPs demonstrated evidence for a secondary association with serum IFN-*α* which was dependent upon the associated autoantibody. Summarizing the four SNPs which demonstrate significant associations following multiple comparison corrections, one SNP is associated with serum IFN-*α* alone, two are associated with autoantibody profiles which are associated with higher IFN-*α*, and one SNP is associated with both serum IFN-*α* and autoantibody profile independently. These relationships are depicted in Figure 5.

### 3.4. SCAN Database Search Results Predicted the Ancestral Background in Which the SLE Phenotype Association Was Observed

The SCAN database search examined both European- and African-derived populations, and the SNPs which were associated with SLE subphenotypes were associated with alternate gene expression in the SCAN database in only one ancestral background. In each of the autoantibody associations, the ancestral background in which the autoantibody association was observed in SLE patients was the same ancestral background in which differential gene expression was observed in the SCAN database (Table 2). The association between rs9568401 and serum IFN-*α* was observed in both ancestral backgrounds, but the SNP was only associated with alternate gene expression in the SCAN database in African ancestry subjects. Overall this general concordance in ancestral backgrounds between the SLE phenotype associations further supports the idea that the SNPs which impact gene expression in human cell lines are more likely to be associated with molecular phenotypes in human disease. Representative transcripts that were differentially regulated by each associated SNP in the SCAN database are also shown in Table 2. 

### 3.5. Case-Control Analysis Does Not Show Large Differences in Allele Frequencies When Comparing All SLE Patients to Controls

As shown in Table 3, we did not observe significant case-control associations for any of the 11 studied SNPs which would withstand statistical correction for multiple comparisons (all *P* > 4.5 × 10^−3^). The initial GWAS was designed to detect associations with either autoantibodies or serum IFN-*α*, and the SNPs we followed up were most strongly associated with these traits. The lack of strong case-control associations at the same SNPs supports the idea that the genetic effects we observe are relevant to patient subsets, and that the power to discover these SNPs would be more limited in a standard case-control study design. 

## 4. Conclusions

In this study, we identify novel genetic variants associated with molecular phenotypes in SLE in two different ancestral backgrounds, using gene expression data as a guide for rational candidate gene selection from a previous GWAS study. In published overall case-control studies of SLE to date, there are examples of both shared associations across ancestral backgrounds [29], and associations which are particular to one or a few ancestral backgrounds [20, 30, 31]. In our study, it is striking that we did not find many associations which were shared between ancestral backgrounds and the majority were distinct to one ancestral background, despite studying molecular phenotypes which are shared across ancestral backgrounds. This would support the hypothesis that while similar molecular pathways may be dysregulated in SLE patients of different ancestral backgrounds, the particular steps in that pathway which are dysregulated may differ by ancestry. These differences would be important to appreciate as we envision personalized therapy using agents which target these pathways, such as the category of anti-IFN-*α* drugs which are being developed for SLE currently. Presumably many autoimmune disease risk alleles which are common in the population have been maintained due to some benefit in increasing immune responses in response to pathogens. Infectious disease has been a major selective force in human history, and it seems likely that different world populations may have developed and selected for different immune system polymorphisms which could result in a similar end pathway output. A striking example of this type of human convergent evolution has been shown in the case of the human lactase gene [32], in which lactase persistence in adulthood was conferred by a number of different polymorphisms that had arisen separately in different world populations, converging upon a similar end pathway result.

Heterogeneity is not unexpected in SLE, as clinically the syndrome is very diverse. Overall case-control genetic studies are likely to be significantly limited due to heterogeneity, as different polymorphisms will be more or less relevant in different patient groups. In the case of physical phenotypes, a number of studies support the idea that different genetic variants will be associated with particular clinical disease manifestations, such as rash, renal disease, and others [33–36]. Diversity in autoantibody and cytokine phenotypes between SLE patients is also well recognized [4, 37, 38]. In this study we examine these two molecular phenotypes and find genetic associations which are relevant to subgroups of patients defined by these molecular characteristics. We have previously demonstrated strong subsetting of genetic risk related to molecular phenotypes in SLE in the case of the IRF5 gene. The majority of the genetic risk of SLE related to IRF5 was found within a particular serologic subgroup which constituted 40% of the overall SLE patient group studied [14]. This gene had been well validated as an SLE-risk gene in previous overall case-control studies [39, 40], but was later shown to have a very strong subgroup effect [14]. It seems likely that this phenomenon will be more widespread, and that many genetic loci could be very difficult to discover in an overall unstratified case-control study. Other autoimmune diseases such as rheumatoid arthritis have already set a strong precedent for the importance of serologic subsets in genetic analysis. The anti-CCP antibody positive versus negative groups of rheumatoid arthritis patients demonstrate large differences in genetic association, including the HLA region [41]. The genes we report in this study have not been previously identified in case-control studies, and in our case-control analysis of these loci support we do not see large overall allele frequency differences. This does not mean that the loci are irrelevant, as they clearly impact important pathogenic subphenotypes in SLE. Instead, this supports the idea that “all cases versus all controls” study designs will have limits, and it is unlikely that we will be able to fully map the genetic architecture of complex diseases fully using only case-control designs, even if very large and well-powered cohorts are used. In summary, it seems likely that both physical or clinical phenotypes as well as molecular phenotypes will need to be incorporated in genetic study designs to address disease heterogeneity and enable continued genetic discovery in autoimmune disease. 

Another benefit of including molecular subphenotypes and gene expression into genetic association studies is that the genetic loci discovered in this manner are immediately linked to some biological alteration. This is especially useful when genes which have not been previously studied are implicated, or if a particular associated genetic variant is not within or near a known gene. If these variants are found in an overall case-control analysis, it can be difficult to plan follow-up functional experiments if little is known about the function of that gene. In our study, we found SNPs which were not in obviously relevant genetic regions, but nonetheless impacted upon important molecular phenotypes and altered expression of immune system molecules. While we cannot know the mechanism by which the genetic variant impacts upon gene expression via our current study, these questions can be followed up and validated in functional experiments.

## Figures and Tables

**Table 1:** Summary of SNPs associated with autoantibody traits.

SNP	Chr.	Nearby Gene	Ancestry	Autoantibody	Odds ratio	*P* value
rs9521996 C	13	ANKRD10	AA	Anti-RNP	2.01	8.0 × 10^−4^
rs1408806 G	9	TYRP1	EA	Anti-Sm	3.48	1.5 × 10^−3^
rs4894215 G	2	—	EA	Anti-Ro	2.16	2.5 × 10^−3^

SNP: single nucleotide polymorphism, chr.: chromosome, autoantibody: the antibody specificity associated with the particular SNP, odds ratio and *P*-value are calculated from the logistic regression model.

**Table 2:** Summary of the 4 SNPs associated with SLE phenotypes and the SCAN database results regarding ancestral background and representative associated transcripts.

SNP	Chr.	Nearby Gene	SLE association ancestry	Associated phenotype	SCAN ancestry	Representative SCAN transcripts
rs9521996 C	13	ANKRD10	AA	Anti-RNP	YRI	IRF3, MIF
rs1408806 G	9	TYRP1	EA	Anti-Sm	CEPH	CASP3, RIPK1
rs4894215 G	2	None within 200kb	EA	Anti-Ro	CEPH	HLADRB1, HLADQB1
rs9568401 G	13	DLEU2	EA, AA	IFN	YRI	IRAK2, NOD2

SNP: single nucleotide polymorphism, chr.: chromosome, SLE association ancestry: the ancestral background in which the SNP was associated with an SLE phenotype, SCAN ancestry: the ancestral background in which that SNP was associated with alternate gene expression, representative SCAN transcripts: genes which differentially expressed due to genotype at that SNP in the SCAN database; two transcripts of the >15 were chosen for inclusion in this table, with an emphasis on those transcripts with immune system relevance.

**Table 3:** Case control analysis of 11 SNPs tested in this study in each ancestral background.

SNP	African Americans			European Americans		
	MAF	OR	*P* value	MAF	OR	*P*-value
rs9521996 C	0.285	1.02	0.86	0.136	1.46	0.12
rs11199974 G	0.258	0.89	0.44	0.482	1.11	0.56
rs7785392 T	0.473	0.80	0.084	0.612	0.78	0.16
rs9568401 G	0.122	0.74	0.12	0.085	1.04	0.90
rs4892122 G	0.279	1.14	0.32	0.295	1.19	0.39
rs4778708 T	0.407	0.95	0.68	0.268	1.10	0.64
rs1340981 A	0.161	0.92	0.61	0.397	0.88	0.47
rs1408806 G	0.174	0.80	0.19	0.246	0.85	0.44
rs4894215 G	0.358	0.94	0.64	0.430	1.08	0.67
rs1569428 G	0.341	0.70	0.0070	0.430	0.92	0.68
rs1159916 C	0.405	0.74	0.018	0.333	0.88	0.49

SNP: single nucleotide polymorphism, MAF: minor allele frequency in controls, OR: odds ratio, as calculated from the logistic regression model.
